# Supplementary material for: Changes in Digestive Microbiota, Rumen Fermentations and Oxidative Stress around Parturition Are Alleviated by Live Yeast Feed Supplementation to Gestating Ewes
Source: J Fungi (Basel). 2021 Jun 4;7(6):447. doi: 10.3390/jof7060447 (PMC8228133; doi:10.3390/jof7060447)
Supplement: Supplementary file 1 [file jof-07-00447-s001.zip › jof-1183832-supplementary.pdf]

Supplementary material

Changes in digestive microbiota, rumen fermentations and oxidative stress around parturition are alleviated by live yeast feed supplementation to gestating ewes

Lysiane Dunière [1,2], Damien Esparteiro [1,2,§], Yacine Lebbaoui[1,2], Philippe Ruiz[2], Mickael Bernard[4], Agnès Thomas[3], Denys Durand[3], Evelyne Forano [2] and Frédérique Chaucheyras-Durand [1,2,*]

Supplementary tables

Table S1: Composition of the commercial concentrate.

Ingredient	g/kg of concentrate
Corn gluten feed	179.7
Rapeseed cake	150.0
Linseed extruded supplement	146.3
Wheat bran	127.0
Barley grain	100.0
Cereal by products	62.0
Corn meal	50.0
Sugarcane molasse	50.0
Beet pulp	50.0
Wheat grain	45.0
Supplements (lime carbonate, microminerals)	40.0

Table S2: Nutritional composition of the diet offered to the ewes. DM=dry matter; NDF= neutral detergent fiber; ADF= acid detergent fiber.

	Prepartum diet	Postpartum diet
**Good quality meadow hay**		
kg/d/ewe	2	3
Dry matter	0.85	0.85
kg/d/ewe (DM)	1.7	2.55
**Concentrate**		
kg/d/ewe	0.8	0.6
Dry matter	0.875	0.875
kg/d/ewe (DM)	0.7	0.525
Forage / Concentrate (on DM basis)	71/29	83/17
**Daily supply (kg/d/ewe)**		
NDF	1.2693	1.7412
ADF	0.6817	0.9512
Crude protein	0.3174	0.3732
Fat (from concentrate)	0.0351	0.0263
Starch + sugars (from concentrate)	0.2156	0.1617

Table S3: qPCR targets and primers.

Microbial target	Target gene	Primer sequence	Ref	Material used for standard curve
Total bacteria	16S rDNA	5′-AGCAGCCGCGGTAAT-3′<br>5′-CAGGGTATCTAATCCTGTT-3′	[17]	Mix of DNA from 11 bacterial strains
Anaerobic fungi	ITS1	5′-GAGGAAGTAAAAGTCGTAACAAGGTTTC-3′<br>5′-CAAATTCACAAAGGGTAGGATGATT-3′	[17]	Cloned amplicon ITS1
Methanogenic Archaea	*mcrA*	5′-GGTGGTGTMGGATTCACACARTAYGCWACAGC-3′<br>5′-TTCATTGCRTAGTTWGGRTAGTT-3′	[16]	*M. wolinii* 87-7
Protozoa	18S rDNA	5′-GCTTTCGWTGGTAGTGTATT-3′<br>5′-CTTGCCCTCYAATCGTWCT-3′	[17]	Cloned amplicon 18S
Fibrobacter succinogenes	16S rDNA	5′-GTTCGGAATTACTGGGCGTAAA-3′<br>5′-CGCCTGCCCCTGAACTATC-3′	[17]	*F.succinogenes* S85
Ruminococcus albus	16S rDNA	5′-CCCTAAAAGCAGTCTTAGTTCG-3′<br>5′-CCTCCTTGCGGTTAGAACA-3′	[16]	*R albus* 7
Ruminococcus flavefaciens	16S rDNA	5′-CGAACGGAGATAATTTGAGTTTACTTAGG-3′<br>5′-CGGTCTCTGTATGTTATGAGGTATTACC-3′	[17]	*R. flavefaciens* c94
Saccharomyces cerevisiae	26S rDNA	5′-AGGAGTGCGGTTCTTTG-3′<br>5′-TACTTACCGAGGCAAGCTACA-3′	[69]	*S. cerevisiae* CNCM I-1077
Faecalibacterium prausnitzii	16S rDNA	F: 5′-GGAGGAAGAAGGTCTTCGG-3′<br>R: 5′-AATTCCGCCTACCTCTGCACT-3′	[25]	*F. prausnitzii* DSMZ 17677
Escherichia coli	16S rDNA	5′-GGAAGAAGCTTGCTTCTTTGCTGAC-3′<br>5′-AGCCCGGGGATTTCACATCTGACTTA-3	[25]	*E. coli* K12
Megasphaera elsdenii	16S rDNA	5′- AGATGGGGACAACAGCTGGA-3′<br>5′-CGAAAGCTCCGAAGAGCCT-3′	[70]	*M. elsdenii* T81

Table S4: rDNA region and primers used for Illumina sequencing.

Types	rDNA region	Fragment length (bp)	Primer name	Primer sequence (5′-3′)
Bacterial 16S	V3-V4	466	341F	CCTAYGGGRBGCASCAG
			806R	GGACTACNNGGGTATCTAAT
Fungal 18S	V4	350	528F	GCGGTAATTCCAGCTCCAA
			706R	AATCCRAGAATTTCACCTCT

Table S5: Q-PCR results for the targeted microbial groups or species in the fecal samples. BS= before

Log_{10} of target gene copy numbers/g feces	BS		Pa		PP	
**Target**	**Control**	**SC**	**Control**	**SC**	**Control**	**SC**
Total bacteria	11.37 ±0.40	11.54 ±0.53	11.51 ±0.38	11.77 ±0.14	11.46 ±0.44	11.54 ±0.39
Escherichia coli	9.02 ±1.31	8.79 ±0.76	8.55 ±1.28	8.94 ±0.53	8.17 ±0.94	8.58 ±0.29
Faecalibacterium prausnitzii	8.28 ±0.41	8.43 ±0.49	8.4 ±0.32	8.61 ±0.23	8.4 ±0.47	8.39 ±0.41
Fibrobacter succinogenes	5.94 ±0.39	6.32 ±1.43	6.15 ±0.69	6.45 ±2.35	6.23±0.00	6.19 ±1.03
Methanogenic Archaea	8.12 ±0.39	8.16 ±0.38	8.27 ±0.29	8.24 ±0.18	8.07 ±0.22	8.21 ±0.34
Anaerobic fungi	4.41 ±0.41	4.89 ±0.91	4.4 ±0.78	5 ±0.94	4.67 ±0.71	4.93 ±1.12
S. cerevisiae	5.31 ±0.32	5.6 ±0.4	5.81 ±0.31	7.34 ±0.87	5.55 ±0.33	5.79 ±0.36

supplementation of SC, Pa=just before parturition, PP= 2 weeks postpartum.

Supplementary figures

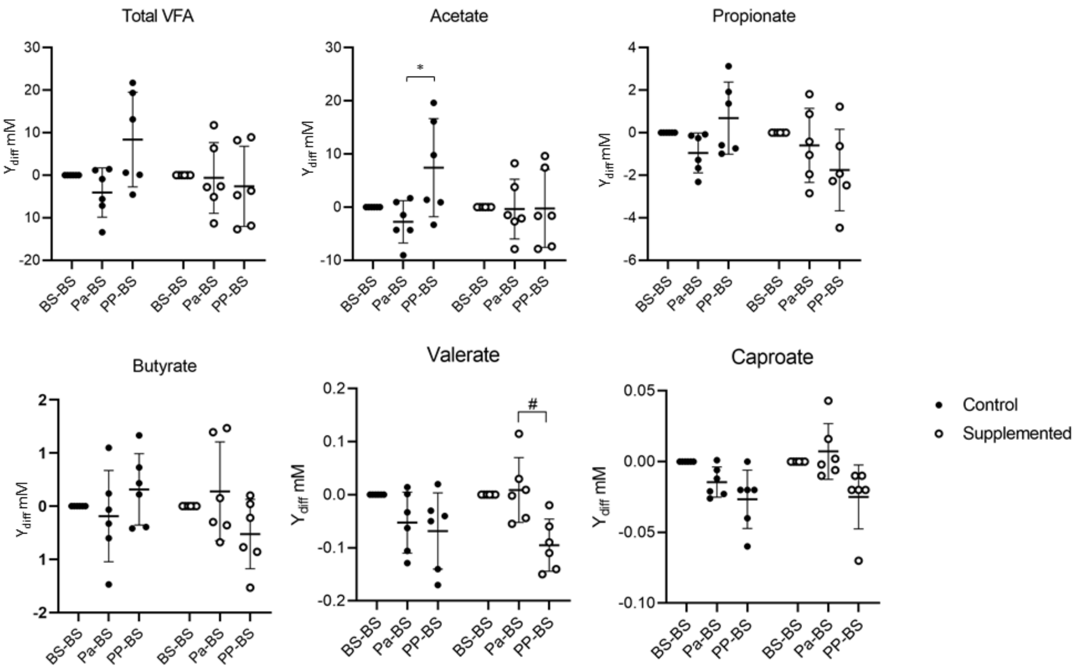

Figure S1: Y_{diff} values at BS, Pa and PP (Mean ± SD) for total VFA, Acetate, Propionate, Butyrate, Valerate and Caproate (delta mM) in Control or SC groups (n = 6) in rumen. Multiple comparisons with Sidak's correction are indicated in the figure only for fixed factors Time or Group with # $p < 0.1$, * $p < 0.05$, ** $p < 0.01$ and *** $p < 0.0001$.

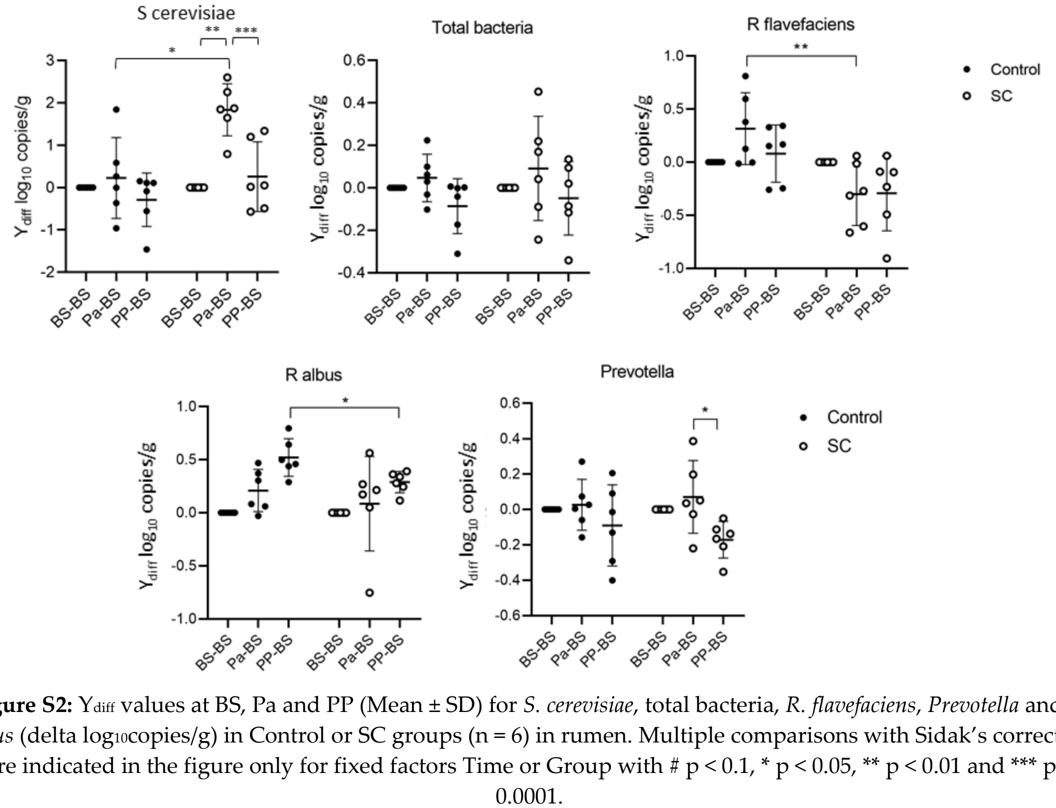

Figure S2: Y_{diff} values at BS, Pa and PP (Mean ± SD) for *S. cerevisiae*, total bacteria, *R. flavefaciens*, *Prevotella* and *R. albus* (delta log_{10}copies/g) in Control or SC groups (n = 6) in rumen. Multiple comparisons with Sidak's correction are indicated in the figure only for fixed factors Time or Group with # $p < 0.1$, * $p < 0.05$, ** $p < 0.01$ and *** $p < 0.0001$.

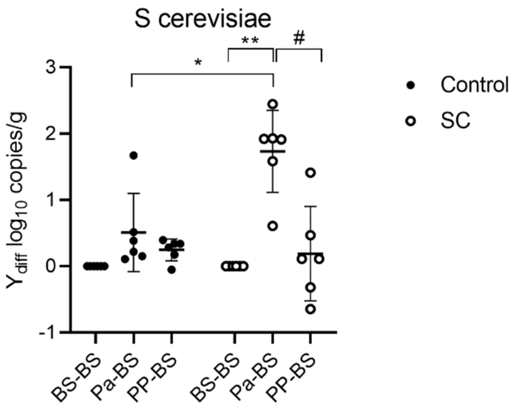

Figure S3: Y_{diff} values at BS, Pa and PP (Mean ± SD) for *S. cerevisiae* (delta log_{10}copies/g) in Control or SC groups (n = 6) in feces. Multiple comparisons with Sidak's correction are indicated in the figure only for fixed factors Time or Group with # p < 0.1, * p < 0.05, ** p < 0.01 and *** p < 0.0001.

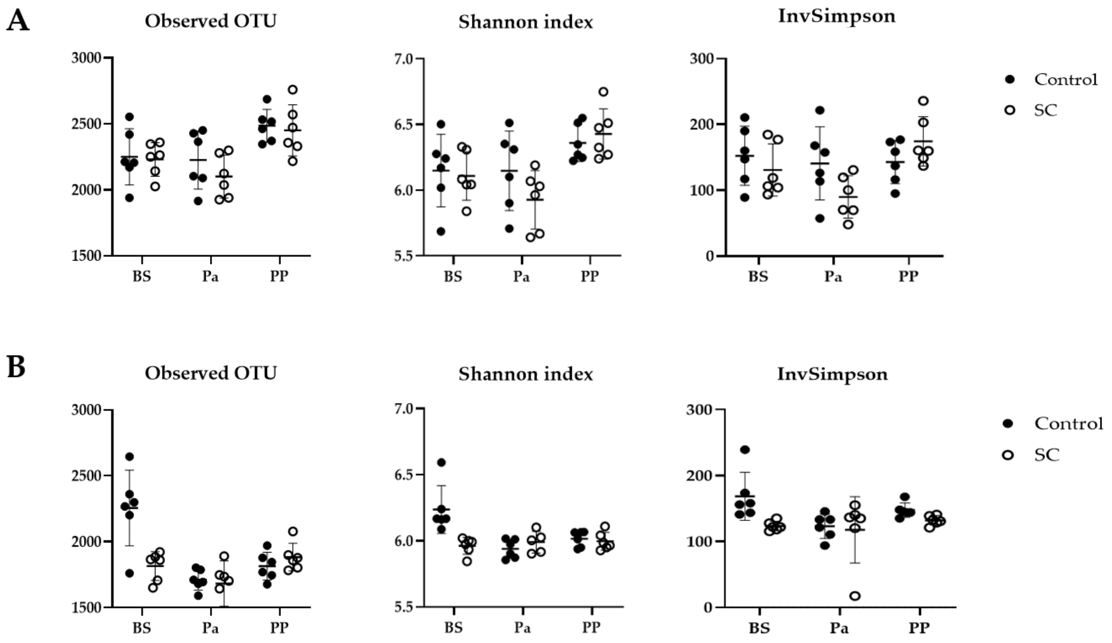

Figure S4: Alpha diversity measures for bacterial and archaeal communities in A) ruminal samples and B) fecal samples. BS= before supplementation of SC, Pa= close to parturition, PP= 2 weeks postpartum.

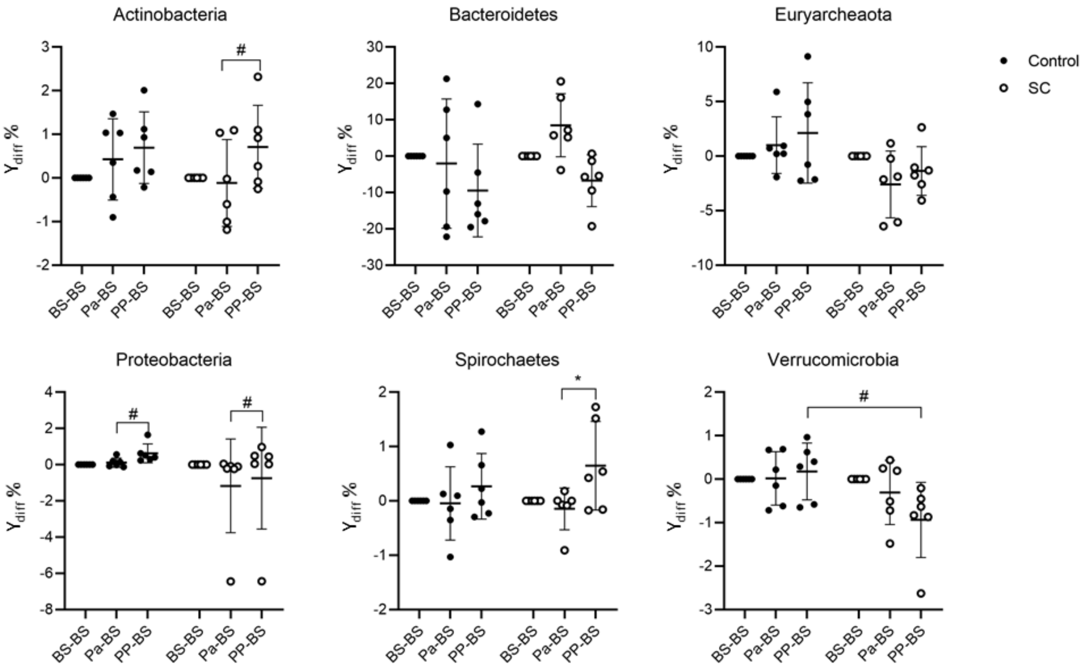

Figure S5: Y_{diff} values at BS, Pa and PP (Mean ± SD) for Actinobacteria, Bacteroidetes, Euryarchaeota, Proteobacteria, Spirochaetes and Verrucomicrobia (delta %) in Control or SC groups (n = 6) in rumen. Multiple comparisons with Sidak's correction are indicated in the figure only for fixed factors Time or Group with # p < 0.1, * p < 0.05, ** p < 0.01 and *** p < 0.0001.

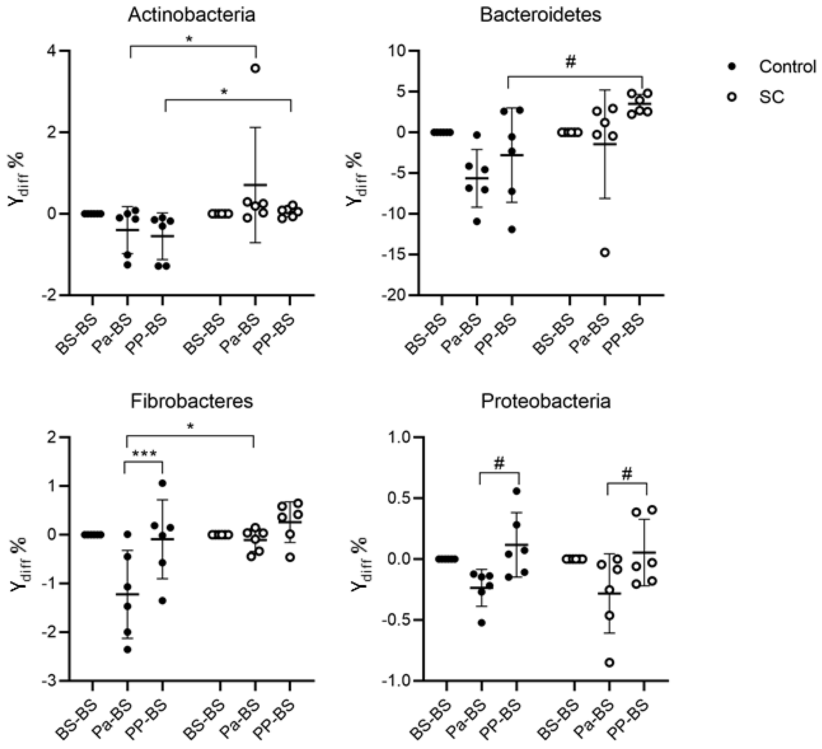

Figure S6: Y_{diff} values at BS, Pa and PP (Mean ± SD) for Actinobacteria, Bacteroidetes, Fibrobacteres and Proteobacteria (delta %) in Control or SC groups (n = 6) in feces. Multiple comparisons with Sidak's correction are indicated in the figure only for fixed factors Time or Group with # p < 0.1, * p < 0.05, ** p < 0.01 and *** p < 0.0001.

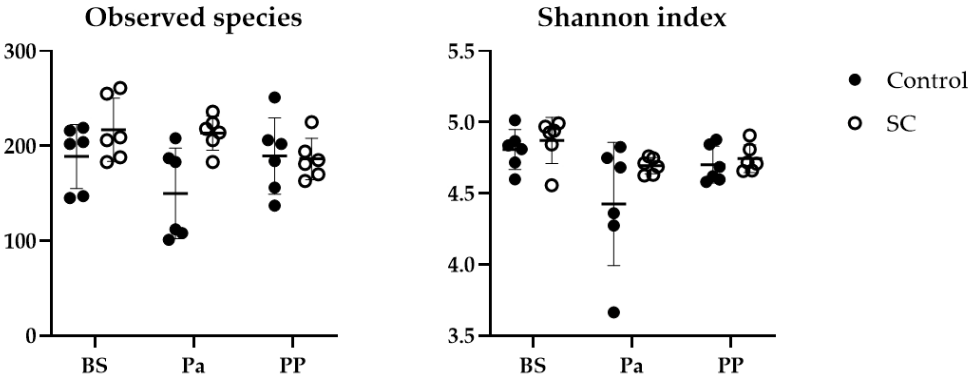

Figure S7: Alpha diversity measures for eukaryotic communities in rumen samples. BS= before supplementation of SC, Pa= close to parturition, PP= 2 weeks postpartum.

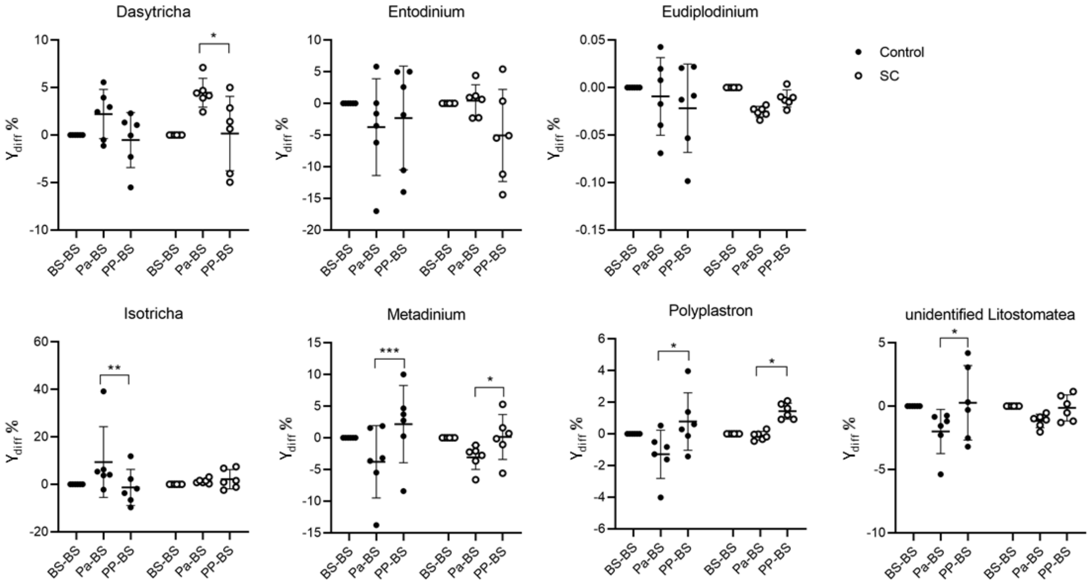

Figure S8: Y_{diff} values at BS, Pa and PP (Mean ± SD) for *Dasytricha*, *Entodinium*, *Eudiplodinium*, *Isotricha*, *Metadinium*, *Polyplastron* and unidentified Litostomatea (delta %) in Control or SC groups (n = 6) in rumen. Multiple comparisons with Sidak's correction are indicated in the figure only for fixed factors Time or Group with # $p < 0.1$, * $p < 0.05$, ** $p < 0.01$ and *** $p < 0.0001$.

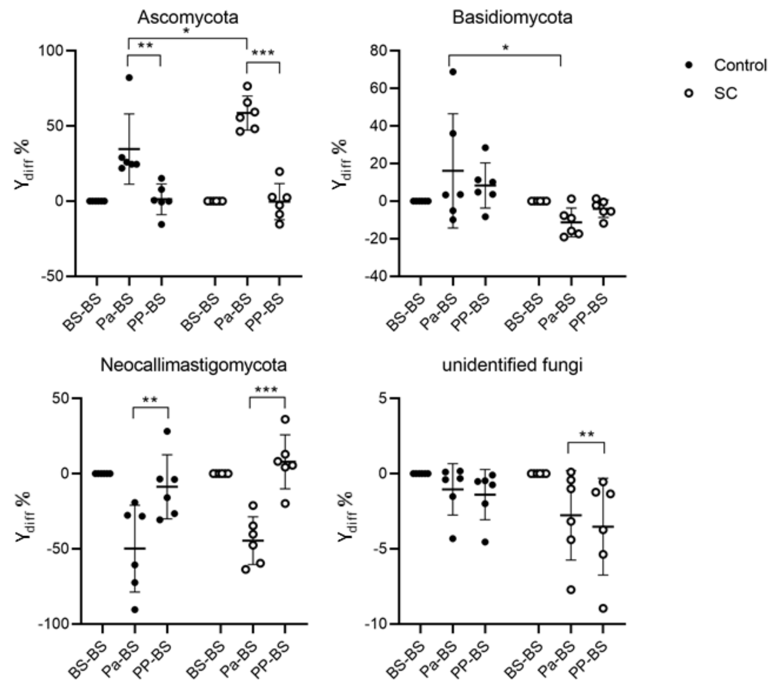

Figure S9: Y_{diff} values at BS, Pa and PP (Mean ± SD) for Ascomycota, basidiomycota, Neocallimastigomycota and unidentified fungi (delta %) in Control or SC groups (n = 6) in rumen. Multiple comparisons with Sidak's correction are indicated in the figure only for fixed factors Time or Group with # $p < 0.1$, * $p < 0.05$, ** $p < 0.01$ and *** $p < 0.0001$.

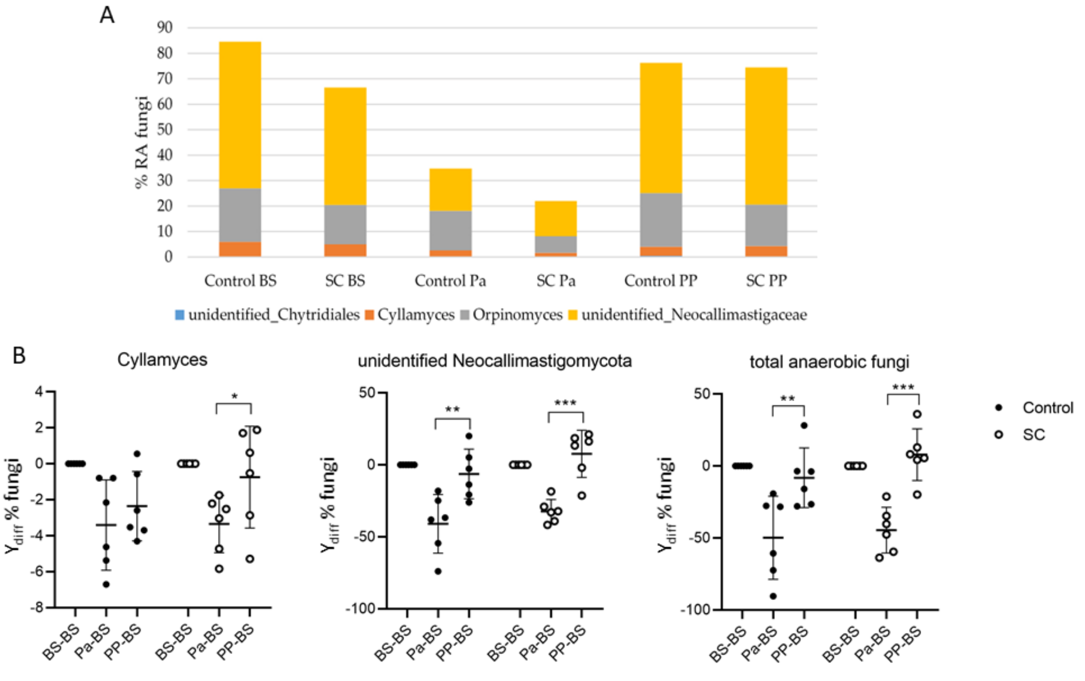

Figure S10: A: Relative abundance (% of total fungal population relative abundance) of anaerobic fungi observed in ruminal samples of Control and SC groups at BS, Pa and PP. **B:** Y_{diff} values at BS, Pa and PP (Mean ± SD) for *Cyllamyces*, unidentified Neocallimastigomycota and total anaerobic fungi (delta %) in Control or SC groups (n = 6) in rumen. Multiple comparisons with Sidak's correction are indicated in the figure only for fixed factors Time or Group with # $p < 0.1$, * $p < 0.05$, ** $p < 0.01$ and *** $p < 0.0001$.
